# UVI31+ Is a DNA Endonuclease That Dynamically Localizes to Chloroplast Pyrenoids in *C. reinhardtii*

**DOI:** 10.1371/journal.pone.0051913

**Published:** 2012-12-17

**Authors:** Manish Shukla, Renu Minda, Himanshu Singh, Srikanth Tirumani, Kandala V. R. Chary, Basuthkar J. Rao

**Affiliations:** 1 Department of Biological Sciences, Tata Institute of Fundamental Research, Colaba, Mumbai, India; 2 Department of Chemical Sciences, Tata Institute of Fundamental Research, Colaba, Mumbai, India; University of Hyderabad, India

## Abstract

UVI31+ is an evolutionarily conserved BolA family protein. In this study we examine the presence, localization and possible functions of this protein in the context of a unicellular alga, *Chlamydomonas reinhardtii.* UVI31+ in *C. reinhardtii* exhibits DNA endonuclease activity and is induced upon UV stress. Further, UVI31+ that normally localizes to the cell wall and pyrenoid regions gets redistributed into punctate foci within the whole chloroplast, away from the pyrenoid, upon UV stress. The observed induction upon UV-stress as well as the endonuclease activity suggests plausible role of this protein in DNA repair. We have also observed that UV31+ is induced in *C. reinhardtii* grown in dark conditions, whereby the protein localization is enhanced in the pyrenoid. Biomolecular interaction between the purified pyrenoids and UVI31+ studied by NMR demonstrates the involvement of the disordered loop domain of the protein in its interaction.

## Introduction

*C. reinhardtii*, a unicellular green alga, undergoes apoptosis in response to UV-C irradiation [1]. In order to understand the process of UV mediated apoptosis in *C. reinhardtii*, we undertook an *in-silico* global genome analysis, which revealed the presence of a UV inducible gene, *UVI31*+ from *C. reinhardtii* (C_2020005). This gene showed strong homology with a similar gene from *S. pombe*. UV inducible gene, *UVI31*+ was first identified in fission yeast *S. pombe. UVI31*+ gene was shown to get up regulated by about 5 to 10 fold within an hour of UV treatment (120 J/m^2^)of the cells [2]. However its expression was unaltered by other DNA damaging or cytotoxic agents, and has no significant homology to the known DNA repair genes [3]. During normal cell cycle, *uvi31*+ transcript increases during G1 phase before septation and also increases during diauxic shift. A null mutant of *UVI31*+ in *S. pombe* showed sensitivity to UV-light, defects inseptation and cytokinesis during the resumption of cell division from the UV damage-induced cell cycle arrest [4].

Protein sequence analyses revealed the presence of a ubiquitous BolA domain, rendering UVI31+ as a member of the BolA protein family. This family consists of the morphogene *bolA* from *E. coli* and its various homologs, which are ubiquitous and conserved from prokaryotes to eukaryotes including humans. Biological function of BolA domain in higher eukaryotes including humans is largely unknown. It is very likely that such conserved domain might be involved with diverse cellular functions depending up on its context. Commonly, BolA proteins have a helix turn helix motif, which is a major structural motif with an ability to bind DNA [5]. Further, most of the members of the BolA family are annotated as secretory proteins [6]. In *E. coli*, *bolA* transcript level increases in response to general stress [7] where the protein has the ability to cause osmotically stable round cells [8] and promote biofilm formation when over expressed [9]. In addition, cells lacking *bolA* do not undergo shape alteration in nutrient restrictive poor medium (M9 medium) at the onset of stationary phase or in response to stress as compared to the wild type cells [8]. On the other hand, BolA protein of *P. fluorescens* is implicated in the metabolism of sulphur containing amino acids and has no effect on bacterial cell morphology and biofilm formation, unlike *E. coli* BolA protein [10].

Here, we report that *UVI31*+ is a UV and dark inducible gene, which gets differentially regulated, during the light-dark (12 hr:12 hr) cycle of *C. reinhardtii*. Interestingly, purified UVI31+ protein as well as endogenous protein from *C. reinhardtii* cells is endowed with DNA endonuclease activity and causes about 1000 fold higher resistance to UV in *E. coli* cells over expressing UVI31+ protein. The protein gets localized in the cell wall and pyrenoid compartments of *C. reinhardtii* cell, the endonuclease activity is retained in these sites. Pyrenoids are the sub-organellar structures in the chloroplast of algae, which specialize in carbondioxide concentration and fixation during photosynthesis in the cell. It has been shown that Pyrenoids contain DNA [11] and are also associated with RNA processing in the cell [11], [12]. Further, UVI31+ gets redistributed into punctate foci within the whole chloroplast, away from the pyrenoid, upon exposure to UV. Biomolecular interaction between the purified pyrenoids and UVI31+ studied by NMR demonstrates the involvement of the disordered loop domain of the protein in its interaction. This result can rationalize localization changes involving dynamic re-association of UVI31+ protein with pyrenoid in *C. reinhardtii* cells.

## Results

### *UVI31*+, a UV and Dark Inducible Gene from *C. reinhardtii*

**Figure 1:**
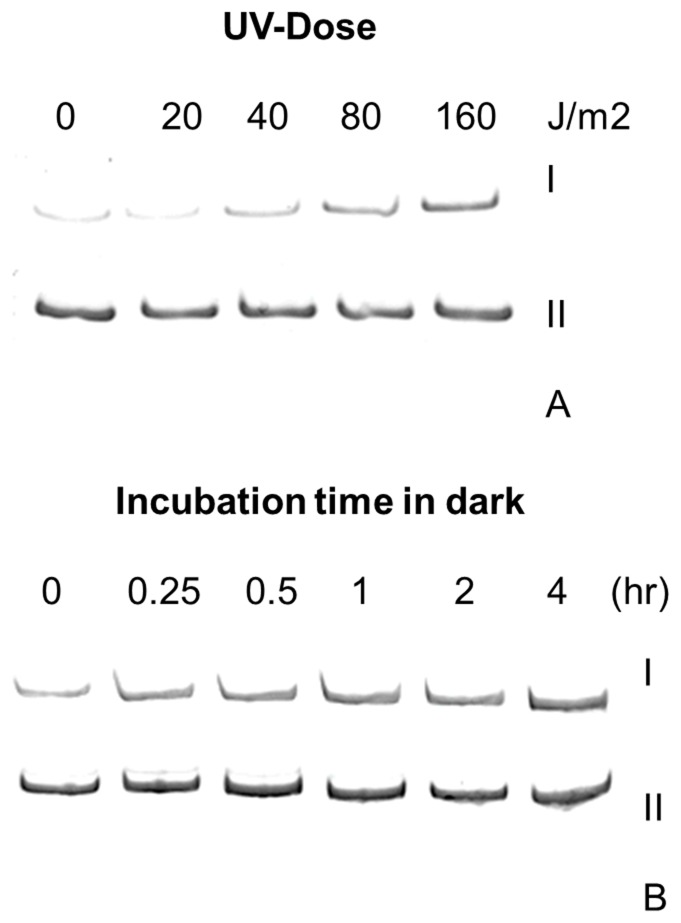
Expression of *UVI31*+ gene from *C. reinhardtii* using semi quantitative RT-PCR. (A) Levels of *UVI31*+ (I) and actin (II) cDNA in control and UV-C irradiated cells. (B) Levels of *UVI31*+ (I) and actin (II) cDNA in control and dark incubated cells.

Our *in-silico* global genome analysis had revealed the presence of a UV inducible, UVI31+ protein from *C. reinhardtii* (XP_001702905) that showed strong homology with a similar gene from *S. pombe (*CAB_16898.1*)* (Table S1). We observed that there were three distinct homologies (*UVI31*+ A gene ID 5728473, *UVI31*+ B gene ID 5720138, *UVI31*+ C gene ID 5716816) of *UVI31*+ in *C. reinhardtii* genome. The *UVI31*+ transcript level in *C. reinhardtii* increased as a function of UV-C fluence and incubation of cells in dark (Figure 1). There was about 12-fold increase in the transcript level when the cells were exposed to UV-C (160 J/m^2^) as compared to unexposed control. In addition, incubation of *C. reinhardtii* in dark also led to induction of *UVI31*+ by about 6-fold. The *C. reinhardtii* actin gene was used as an internal control to assess the *UVI31*+ transcript levels semi-quantitatively. The cloned gene corresponding to experimentally detected UV and dark induced transcript matched fully with the homologue U*VI31*+ A, both at DNA and protein sequence level. This gene (1063 bp) comprises of three introns and four exons, encodes a transcript of 303 bases and a protein of 100 amino acid length (Protein ID-XP_001702905.1). We searched for upstream regulatory elements of *UVI31*+ gene that are relevant for DNA damage response and cell cycle control. The analysis revealed a TATATAA box-like signature at −375 position, a DNA damage responsive like element (DRE) TCTTGAA at −70, two Mlu1 cell cycle box (MCB) like elements (AGGCGC and TCGTGA) at −793 and −646 positions respectively and two elements containing signature sequences of SWI4/6 dependent cell cycle box (SCB) (GACAA and AAAGAAAA) at −738 and −181 respectively. Such DRE-like sequences are found in the upstream of several genes induced during DNA damage response conditions [13]. Furthermore, MCB and SCB-like elements are found in the promoter regions of genes over-expressed in *S. cerevisiae* during the late G1 phase of cell cycle [3]. The sequence signatures such as DRE, MCB and SCB are consistent with a gene that is DNA damage inducible and cell cycle regulated, expected of known phenotypes of *UVI31*+ gene homologue in *S. pombe* [3].

Interestingly, *in silico* search for similar motifs showed that the sequence of *UVI31*+ has a BolA-like domain from 16^th^ to 100^th^ amino acid residues. This prompted us to align different BolA and BolA-like sequences from various organisms with the *C. reinhardtii* UVI31+. The percentage of amino acid residue identity was the highest with *C. albicans* (48%) and the least with *E. coli* BolA protein (27%), thus ascribing this protein as being closely related to the fungal proteins (Table S1).

### UVI31+ Protein of *C. reinhardtii* Reveals Properties of Both *E. coli* BolA and UVI31+ of *S. pombe*

**Figure 2:**
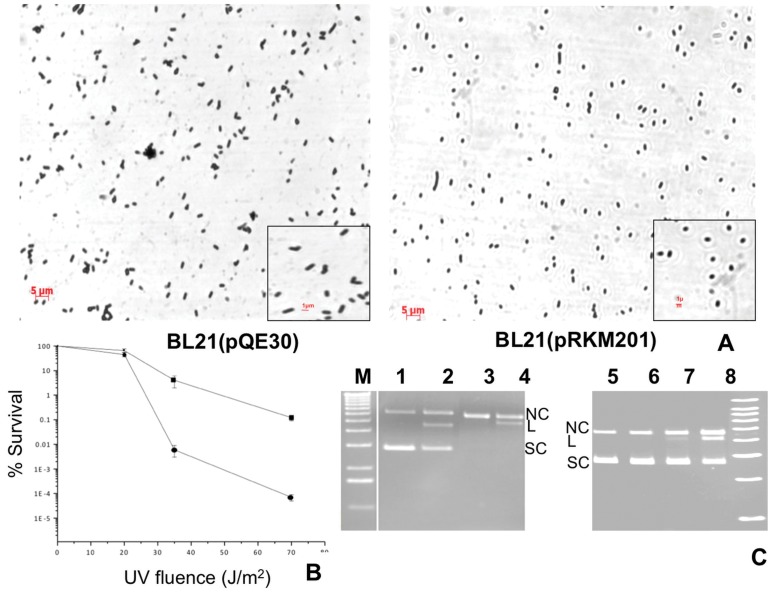
UVI31+ overexpression physiology in E. coli and characterization of its endonuclease activity. (A) Morphology of *E. coli* BL21 cells. Phase contrast micrographs of BL21 (pQE30); and BL21 over expressing UVI31+ (pRKM201). (B) UV survival of *E. coli* BL21 strains harboring the plasmid pRKM201 over expressing UVI31+ (▪)and pQE30 (•). (C) Endonuclease activity of UVI31+.Plasmid DNA un-irradiated (lane 1) or irradiated with 7.2 J/m^2^ of UV-C (lane 3) was incubated with 13 µM of UVI31+ respectively (lanes 2 and 4), for 30 min at 37°C. Plasmid DNA (lane 5) was incubated with 13 µM of UVI31+ (lanes 6–8) for 5, 15 and 30 min, respectively. M, 1 kb DNA ladder.

Over expression of *bola* gene in *E. coli* causes formation of osmotically stable spherical cells [8]. In spite of low sequence homology (identity: 27% and similarity: 54%), *C. reinhardtii* UVI31+ protein shows substantial structural homology with the known tertiary structure of *E. Coli* BolA ([14] and unpublished observations). With this in mind and gain an insight into the BolA domain of UVI31+, we tested whether UVI31+ also causes round morphology in bacterial cells. Our data suggests that *E. coli* cells harboring the plasmid pRKM201 that over expresses UVI31+ protein are predominantly round in shape (65%; Fig. 2A) as opposed to *E. coli* cells that harbor control vector (pQE30) lacking *UVI31*+ insert (27%; Figure 2A). Further, UVI31+ protein expressing round cells showed normal growth kinetics and colony forming units.

The sequence homolog of *UVI31*+ in *S. pombe* is specifically induced by UV stress [2] and its null mutant is also sensitive to UV-light [15]. We tested *E. coli* BL21 (DE3) cells over expressing *C. reinhardtii* UVI31+ protein for its UV-sensitivity phenotype. Cells over expressing UVI31+ protein showed ∼1000 fold more resistance to UV in the range of 35 to 70 J/m^2^ compared to cells harboring only the control vector (pQE30) (Figure 2B). The round cell morphology and UV-resistance conferred by UVI31+ over expression in *E. coli* cells was specific to UVI31+ protein. A control protein (human Translin) over expression does not cause these phenotypes (data not shown).

### Purification and Biochemical Characterization of UVI31+ from *E. coli*

Sequence analysis of the cDNA (303 bp) confirmed the presence of an uninterrupted open reading frame. Recombinant UVI31+ protein was purified from *E. coli* by Ni2+-NTA agarose column, followed by gel filtration chromatography [14]. The protein eluted as a major peak at 87 ml, a volume corresponding to a monomer of approximately 13 kDa. However, a small hump was also observed between 68^th^–75^th^ ml indicating a dimeric form. SDS-PAGE combined with Comassie Brilliant Blue, silver nitrate staining and western blot analysis using anti-Histidine antibody confirmed the purity of the protein eluted (Figure S1). Assessing the molecular mass and protein sequence of the protein elute (13,404±33 Da) by mass spectrometry further confirmed it to be UVI31+.

Most BolA proteins have two conserved basic regions harboring a helix-loop-helix domain predicted to be involved in DNA binding. We therefore checked the DNA binding ability of purified UVI31+ protein. Different plasmids were incubated with UVI31+ pure protein and checked for electrophoretic mobility shift. Surprisingly, we found that UVI31+ showed DNA nicking instead of binding activity (Figure 2C). It acts as a nonspecific endonuclease on both UV-irradiated and un-irradiated supercoiled and nicked circular forms of double stranded plasmid DNA, converting them into linear form. Endonuclease action was also evident in the time-course analyses performed in a separate electrophoretic run (Figure 2C). Under the experimental conditions described here, the DNA did not fragment further. However, when the reaction was performed at higher protein concentrations and/or for prolonged incubation times, we did see additional fragmentation of full-length linear DNA (data not shown). Lack of smearing from full-length linear band suggested relative absence of dsDNA-specific exonuclease activity. The reaction required Mg (II) and was inhibited in the presence of EDTA and was independent of ATP or GTP hydrolysis. Purified protein showed a gel filtration profile that contained monomer and dimer forms. DNA endonuclease activity profile scaled in proportion with protein absorbance and the activity associated with the dimer peak was relatively higher than that of monomer (data not shown). Detailed characterization of DNA endonuclease activity, study of mutations that modulate the same is part of a separate investigation. In this paper, we describe the protein localization and its modulation in *C. reinhardtii* cells, as described below.

### UVI31+ Protein Localization in *C. reinhardtii* Cells

**Figure 3:**
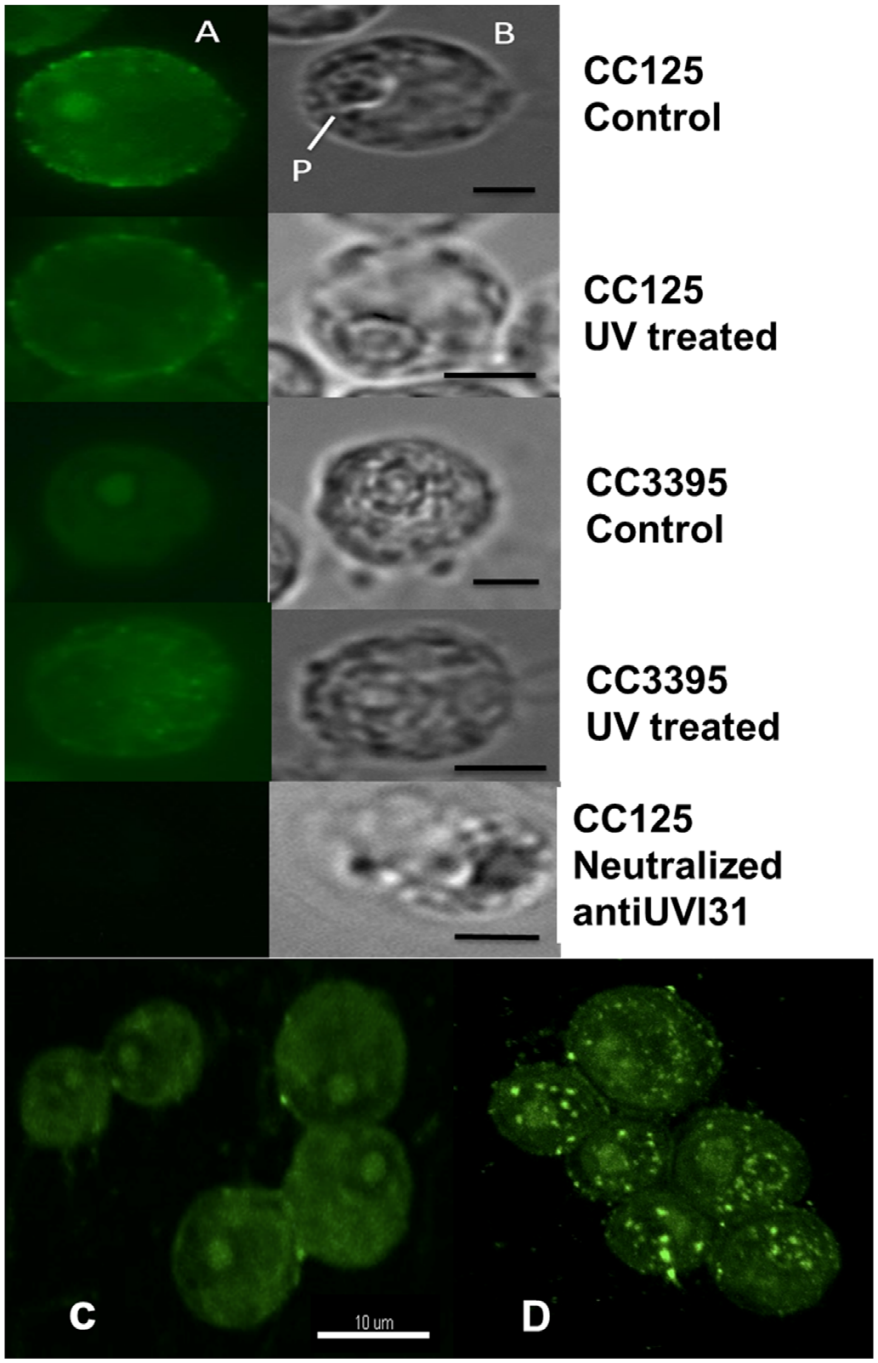
Localisation of UVI31+ protein in *C. reinhardtii* cells. Immunofluorescence staining of UVI31+ protein in *C. reinhardtii* CC125 and CC3395 (from dark phase) and 160 J/m^2^ UV treated cells (A) and bright field images (B) (Bars 5 µm). 3D rendering of UVI31+ immunofluorescence confocal images of CC3395 control (C) and 160j UV treated cells (D).

Polyclonal antibodies were raised against purified UVI31+ protein. Purified antibody preparation as well as control antibodies (neutralized with purified UVI31+ protein) (see Methods) were used to perform immunofluorescence experiments against *C. reinhardtii* cells as well as purified cellular fractions (as described below). A diffuse signal of anti-UVI31+ antibody was seen throughout the cell, but it was more intense in the cell wall and pyrenoid locations (Figure 3). We ascertained the cell wall signal by using a cell wall minus mutant (CC3395) where the peripheral staining was absent. However, this mutant showed localization of UVI31+ in the pyrenoid region, which was identified by bright field image of the cells (Figure 3). Cell wall associated antibody staining was variable in different growth and culture conditions. However pyrenoid staining was more consistent in wild type (CC125) and cell wall minus strain (CC3395). UVI31+ neutralized antiserum showed the loss of signal at pyrenoid as well as cell wall locations. In fact, this control revealed a complete loss of diffuse staining associated with UVI31+ protein in the cells, thereby indicating that antibody staining was specific to UVI31+ protein. Since *UVI31*+ gene is UV inducible, we wanted to check UV-stress associated changes in the cell. At the UV dosage of 160 J/m^2^ (about 30 to 40% cell lethality), we observed a significant loss of the pyrenoid-associated signal in wild type (CC125) and cell wall minus strain (CC3395). However, UV exposure did not affect cell wall associated signal in CC125. Other intracellular distributional changes of UVI31+ protein in CC125 were less distinct. In CC3395 cells (cell wall minus), UV exposure seems to have generated a punctate distribution of UVI31+ protein. In our experience, CC3395 cells were better than CC125 for imaging UVI31+ protein changes. The punctate staining of UVI31+ protein in UV treated CC3395 cells were further corroborated by confocal imaging of cells. Confocal images of immunostained cells (Digital slices in Figure 2) and 3D rendered version of the same (Figure 3C & 3D) revealed that pyrenoid staining is reduced accompanied by increase in punctate staining of UVI31+ in cells that were UV-treated. Punctate staining is visible across all the slices, thereby suggesting that UVI31+ protein spreads throughout the chloroplast following UV-treatment.

### Localization and DNA-endonuclease Activity of UVI31+ Protein in Purified Pyrenoid and Cell Wall Fractions

**Figure 4:**
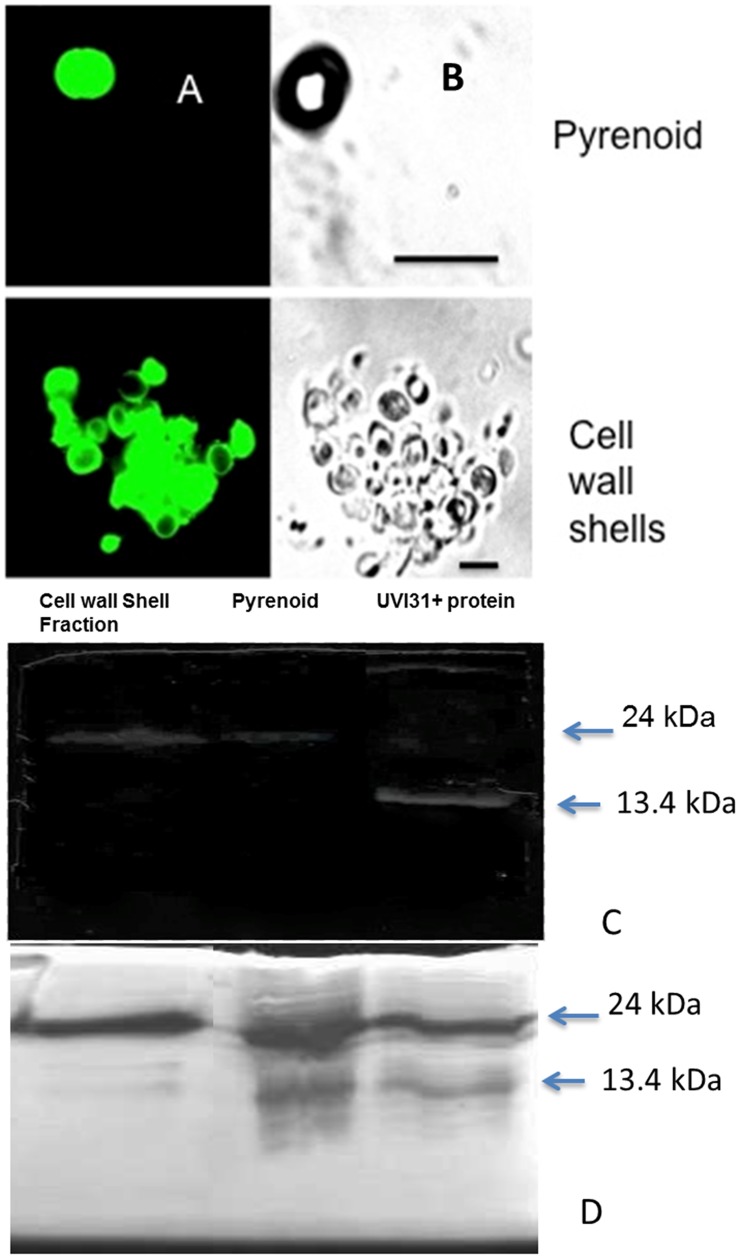
Localization of UVI31+ protein in the pyrenoid and cell wall by antiUVI31+ staining and endonuclease zymogram. Immunofluorescence staining of UVI31+ protein (A) and bright field images (B) in isolated Pyrenoid and cell wall fractions of *C. reinhardtii* CC125. (Bar 5 µm). Anti UVI31+ antibody western blot analysis (C) and zymogram assay of the endonuclease activity associated with UVI31+ protein in the partially purified cell wall and pyrenoid fractions of *C. reinhardtii* (D).

We purified pyrenoid and cell wall fractions according to a standard procedure ([16] and [17], see Methods). The purified fractions showed expected morphological characteristics in bright field imaging: pyrenoid fraction showed spherical bodies of about 3–5 micron size; cell wall fraction revealed largely empty spherical shells, devoid of cell contents (Figure 4A & B). When the fixed preparations of the cell wall and pyrenoid fractions were analyzed by immunofluorescence, bright staining of anti UVI31+ antibodies was seen (Figure 4A & B). This pattern corroborated the staining seen for *C. reinhardtii* cells. Though whole cell protein western showed no signal for antiUVI31+ the purified cellwall and pyrenoid western were positive (Figure S3). Western blot analyses of these fractions were performed along with purified UVI31+ protein as a standard control for comparison. The standard protein lane showed a strong western signal at 13.4 kDa (monomer position) while the same at dimer position was rather weak. However, the dimer specific signal was strong in pyrenoid and cell wall fractions (Figure 4C). Monomer levels were too low to be detected in these cell fractions. Western blot analyses of whole cell extracts (control & UV-treated) had failed to detect UVI31+ protein signal (Figure S3), suggesting that anti-UVI31+ Ab is not only unable to detect endogenous UVI31+ protein but also is unable to spuriously cross-react with any other cellular proteins in the whole cell extract sample, perhaps due to insufficient titer strength of the Ab preparation. However, we succeeded in obtaining the western signal for UVI31+ in samples, which are enriched with this protein (Figure 4D). We therefore conclude that anti-UVI31+ Ab signal in the western blot analyses performed on pyrenoid and cell wall samples is specific.

We wanted to verify whether the protein present in pyrenoid and cell wall fractions also shows DNA endonuclease activity, consistent with the purified UVI31+ protein. DNA endonuclease activity was tested by a zymogram assay, following renaturation of proteins from pyrenoid and cell wall fractions resolved in SDS-PAGE. Both the monomer and dimer forms of the purified UVI31+ protein showed the endonuclease activity, where the dimer was relatively more active compared to monomer (Figure 4D). Interestingly, zymogram assay being more sensitive than western blot enabled us to detect the trace levels of UVI31+ monomers in pyrenoid fractions. This observation corroborated our earlier result where dimeric form of protein from gel-filtration profile had shown higher endonuclease activity (data not shown). Interestingly, the cell wall and the pyrenoid fractions also showed the endonuclease activity in protein bands corresponding to the dimer form. The activity associated with monomer form of the protein was faint, but discernible.

### UVI31+ localization Changes During Light-dark Cycle and Concomitant Effects Following UV-C Exposure

**Figure 5:**
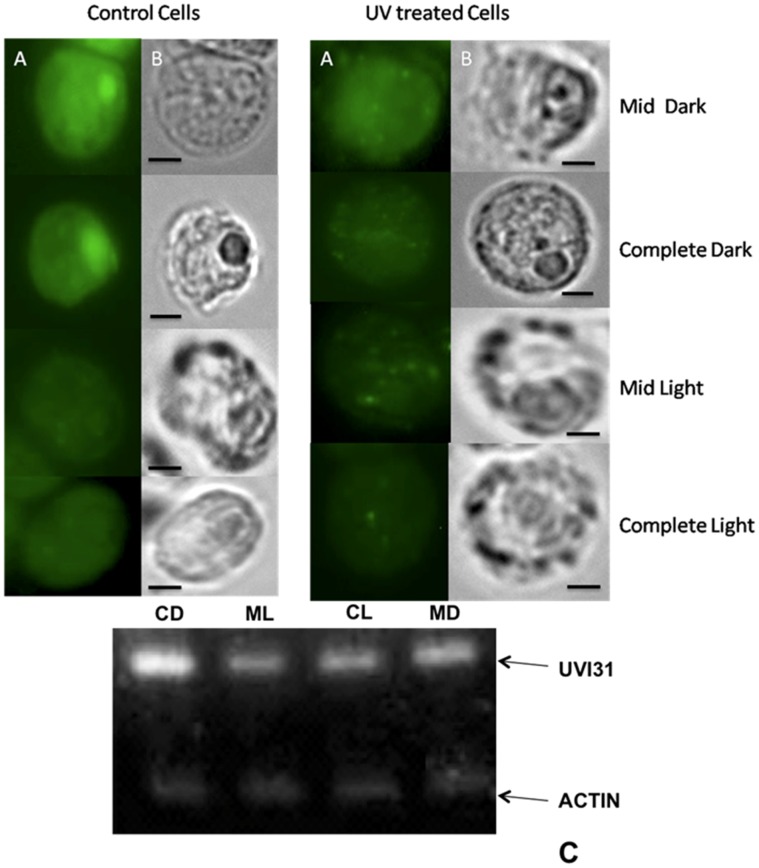
Localisation changes of UVI31+ protein in *C. reinhardtii* cells during light dark regime and UV exposure. Immunofluorescence staining of UVI31+ protein (A) and bright field images (B) of *C. reinhardtii* CC3395 control and UV treated (40 J/m^2^)cells at various intervals of the dark and light regime (Bar 5 µm). Expression analyses of *UVI31*+ gene from *C. reinhardtii* CC3395at various intervals of the dark and light regime by RT-PCR (C).

It is the 12 hr dark period that is central to division processes in *C. reinhardtii* during which UVI31+ protein function is likely to be paramount, as inferred from the studies reported on UVI31+ homologue in *S. pombe* [3], [4], [18]. We therefore set out analyzing changes in *UVI31*+ transcript levels as well as UVI31+ localization in *C. reinhardtii* cells during different time points of growth in synchronized cells from light-dark (12 hr:12 hr) cycles. Experiments were carried out on CC3395 cells grown photoautotrophically in acetate-free TP medium. We performed the immunofluorescence analyses on synchronized cells harvested at four different time points, mid-dark (MD; 6 hr into dark), complete dark (CD; 12 hr into dark), mid-light (ML; 6 hr into light) and complete light (CL; 12 hr into light) (see Methods). Pyrenoid specific location of UVI31+ protein was evident only in dark phase cells (MD & CD, Figure 5A). The imaging resolution does not allow us to discern if there is any punctate pattern in pyrenoid in any conditions. The staining was weak and diffuse, with much reduced localization at pyrenoids, in light phase cells (ML & CL, Figure 5A). UV-C exposure caused changes in protein localization (as shown earlier, Figure 3): pyrenoid specific staining in dark-phase cells was reduced, with concomitant appearance of punctate and diffuse staining in the cell (Figure 5B). Light-phase cells also exhibited similar punctate and diffuse staining following UV-C exposure, where the intensity was less as compared to equivalent dark-phase cells. We believe that the weaker IF signal in UV-treated cells in Figure 5 (compared to that in Figure 1 & 3) is perhaps due to reduced UV-C as well as other differences in the conditions (see Methods). The focus of the experiment described in Figure 5 is on control samples that are not exposed to UV-C, where we see dramatic changes in UVi31+ protein localization at different stages of light-dark cycle. Based on the confocal imaging data described in Fig. 3, we believe that the diffuse and punctate staining of protein observed in light phase cells and UV-treated dark phase cells relate to the spreading of protein into chloroplast compartment. We also carried out RT-PCR analyses of *UVI31*+ transcript level changes. We found that the *UVI31*+ transcripts reach a maximum in CD, followed by a drop through ML/CL and the start of a surge at MD that peaks at CD (Figure 5C).

### UVI 31+ Interacts with Pyrenoids via Specific Structural Domains of the Protein

Prompted by our observations that UVI31+ protein exhibits localization changes that involve dynamic association and dissociation of the protein with pyrenoids in *C. reinhardtii* cells (Figure 3C & D; Figure 5), we tested the ability of purified UVI31+ protein to directly interact with purified pyrenoids, *in vitro*. We used a solution NMR technique to study the biomolecular interaction between the purified pyrenoids and UVI31+. We wanted to probe UVI31+ binding to pyrenoid at amino acid residue level resolution. We eventually intend to map the UVI31+ protein domain that interacts with pyrenoid for designing appropriate genetic perturbations, *in vivo*. This is feasible by mapping the interacting residues using uniformly ^13^C and ^15^N labeled UVI31+ protein complexed with unlabeled pyrenoids. The heteronuclear single quantum coherence/correlation (HSQC) is the most frequently used experiment in such a study. The resulting two-dimensional (2D) spectrum correlates ^1^H spins with its directly attached heteronucleus, an NMR active nucleus such as ^13^C and ^15^N. The spectrum provides residue level information. In such an interaction study, one can isotopically label one of the interacting molecules, leaving the other one without label. Thus, when one records the HSQC spectrum of such a complex, one observes the spectral signatures arising from the isotopically labeled molecule alone, and hence, it turns out to be less complex for analysis. A comparison of the spectral signatures of the labeled molecule in its free state and as part of the complex throws light on the biomolecular interaction. Though the HSQC experiment can be performed using natural abundance of the ^15^N isotope, one normally uses an isotopically labeled protein, which is produced by expressing it in bacterial cells grown in ^15^N-labelled medium. The 2D [^15^N-^1^H] HSQC provides the correlation between the backbone ^15^N and its directly attached amide proton (^1^H^N^) of each residue with an exception of Pro residue, which lacks ^1^H^N^. When a protein interacts strongly with high molecular weight cellular component such as DNA, heat shock protein complex, cell membrane, pyrenoid organelle etc, the bound residues of the protein can cause line broadening up to the complete loss of the respective NMR signals due to their slowing in tumbling motion [19]. Alternately, binding that does not significantly reduce tumbling motion of residues, but alter their chemical environment, leads to chemical shift perturbations [20].

**Figure 6:**
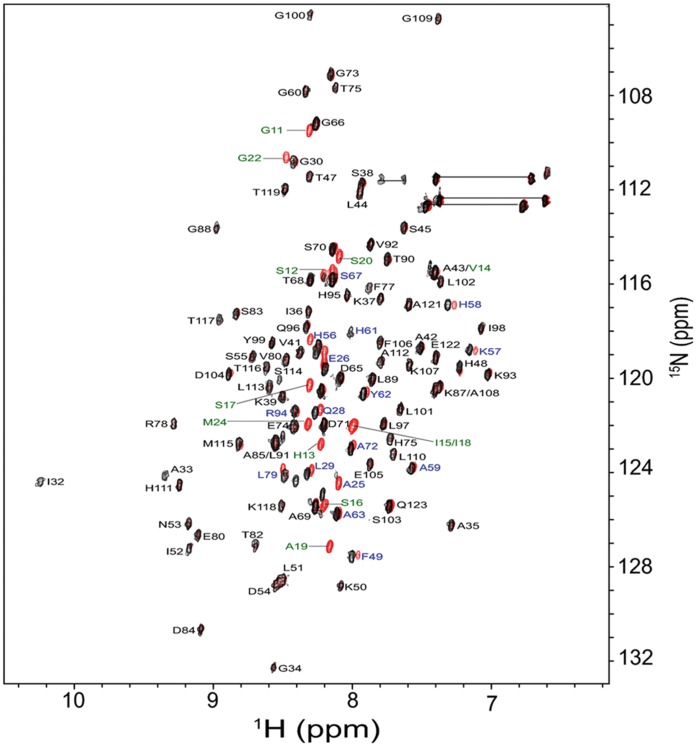
An overlay of 2D [^15^N-^1^H] HSQC spectrum of the complex (formed between u-^15^N-labelled UVI31+ and pyrenoids) (shown in black) with that of u-^15^N-labelled UVI31+ alone (shown in red). Most red spots overlap with black spots in the spectrum (residues that do not change in the complex, also shown as black letter code of the amino acid residues). Signals of a few residues are lost in the complex (shown as green letter code of the amino acid residues). Signals of a few residues are shifted due to chemical shift perturbations (shown as blue letter code of the amino acid residues). The amine-group peaks from the side-chains of Asn and Gln appear as doublets near the top right corner of the spectrum as connected by horizontal lines. The experiments were recorded at pH 6.4 and 298 K. The spectra were recorded on Bruker Avance 800 MHz NMR spectrometer with 256 and 2048 complex points along t_1_ and t_2_ dimensions, respectively.

In the present study, we used 2D [^15^N-^1^H] HSQC to characterize the biomolecular interaction between the purified pyrenoids and uniformly ^15^N-labeled UVI31+ (200 µM). Earlier, we had carried out almost complete sequence specific ^1^H, ^13^C and ^15^N resonance assignments of UVI31+ [14] and the 3D solution structure of the protein was determined by NMR spectroscopy (Manuscript under preparation). With this in the backdrop, when we added the protein to purified pyrenoids fraction, we observed distinct chemical shift perturbations in the HSQC spectrum of the complex (Figure 6). Visual inspection of the spectrum revealed subtle changes in the spectrum. In the complex, some of the original peaks broadened out and some showed distinct changes in their chemical shifts, while many remained unperturbed. Figure 6 shows the overlay of the 2D [^15^N-^1^H] HSQC spectrum of the complex (formed after mixing u-^15^N-labelled UVI31+ with pyrenoids) with that of u-^15^N-labelled UVI31+ alone. The broadened peaks included the spectral signatures of the N-terminal amino acid residues belonging to the entire polypeptide stretch between the residues at positions G11 and M24 (Figure 6). The peaks, which showed substantial perturbations in the chemical shifts, included those of A25, E26, Q28, L29, F49, H56, K57, H58, A59, H61, A63, S67, A72, L79 and R94. Rest of them does not undergo any chemical shift perturbation. UVI31+ protein thus interacts with pyrenoids largely through the segment G11–M24, as evidenced by the missing peaks due to signal broadening (Figure 6). Further, significant chemical shift perturbations seen near the N-terminal polypeptide stretch of the protein (A25, E26, Q28 and L29) and in the disordered loop region of the protein (H56, K57, H58, A59, H61, A63, S67, A72, L79 and R94), which is in a close proximity of the N-terminal polypeptide stretch, support the biomolecular interaction between UVI31+ and pyrenoid. We therefore, conclude that the biomolecular interaction is specific and perhaps is of physiological relevance. That will be tested further by *in vivo* genetic perturbation experiments as a part of separate study. Complete sequence specific ^1^H, ^13^C and ^15^N resonance assignments and 3D structural characterization of the complex formed after mixing u-^15^N-labelled UVI31+ with pyrenoids is in progress.

## Discussion

UVI31+ protein has been shown to be important in regulating the cell division function in *S. pombe*, especially after the cells resume back following cell-cycle arrest [4]. More specifically, the protein seems to negatively regulate septum formation and cytokinesis during the onset of cell division [4]. *UVI31*+ null mutant cells show higher tendency to form spurious septa and undergo enhanced cell proliferation yielding smaller size cells, compared to wild type control cells [4]. In addition, *UVI31*+ transcript levels go up following UV-exposure of cells and show cell growth phase dependent cyclic changes in expression levels where transient increase is seen during diauxic shift of growth phase [3]. In this study, we corroborate the basic features of UVI31+ protein as described above from *S. pombe* in another single celled organism, *C. reinhardtii*, and shed light on some additional features of the protein. After the previous elaboration of *UVI31*+ in *S. pombe*, the only organism where this protein function has been studied so far, our study perhaps represents the second system being investigated to gain more understanding of cellular function of UVI31+ protein. *C. reinhardtii* offers a unique system where cell division is regulated by light-dark (12 hr:12 hr) cycles: cells grow in size, reach “commit to divide” stage after reaching a critical cell size in 12 hr light period followed by 2 or 3 successive divisions yielding 4 or 8 daughter cell clusters in the 12 hr dark period (Harris 2009). It is the 12 hr dark period that is central to division processes in *C. reinhardtii* during which UVI31+ protein function is likely to be paramount.

Firstly, we were encouraged by the observation that *UVI31*+ gene transcript levels go up when the *C. reinhardtii* cells are grown in dark (division phase in *C. reinhardtii)* and also when the cells are exposed to UV-C (Figure 1& 5C), The sequence signatures such as DRE, MCB and SCB that we detected at upstream region of *UVI31*+ gene also add credence to our conclusion that the gene is DNA damage inducible and cell cycle regulated, just as the *UVI31*+ of in *S. pombe* [3]. All these observations, put together, were in line with the lessons learned in *S. pombe* system, which prompted us to study *UVI31*+ function further. In addition, UVI31+ protein from *C. reinhardtii,* when over-expressed in *E. coli* cells, conferred several hundred fold higher resistance to UV-C compared to control cells. Moreover, as in *UVI31*+ gene of *S. pombe*, the *C. reinhardtii* gene also showed a discernible BolA domain sequence (Table S1). Importantly, *E. coli* cells over expressing UVI31+ protein from *C. reinhardtii* conferred round cell morphology in the bacterial cells, a phenotype that is ascribed to BolA domain proteins [8]. Therefore all the hallmarks of UVI31+ protein from *C. reinhardtii* seem to fit the features well described for *S.pombe* protein.

Our current study extends the analyses further, in the context of *C. reinhardtii* biology, as described below. 1. The protein shows dominant localization to cell wall and pyrenoid fractions (Figure 3). 2. Protein localizes to pyrenoids only when the cells are grown in dark (Figure 5A). Contrarily, cells grown in light exhibit distributed localization of UVI31+ protein, not associated with pyrenoids (Figure 5A). 3. Intriguingly, over expressed-purified protein as well as the endogenous protein from *C. reinhardtii* exhibits DNA endonuclease activity. The protein localization to cell wall region has been enigmatic. A comparison using the UVI31+ protein structure solved by solution NMR [21] (Rout, A. K. *et. al.*, Unpublished), revealed that there is considerable structural similarity with *Serratia* family endonuclease structural motifs. Such enzymes with unusual localization might also perform functions other than that in replication, repair and recombination [22] and thereby have roles in host defense [23], apoptosis [24] and cell division [25]. One could speculate that cell wall localization of a nuclease might poise for its release extracellularly in stress responses. Alternately, cell wall localization of UVI31+ protein possibly places it right for rendering it as cell shape reorganizer expected of a BolA domain (morphogene) containing protein.

All these attributes suggest that UVI31+ protein is associated with important functions whose specific relevance is unclear. Even though, we have been yet unsuccessful in either over expressing the protein in *C. reinhardtii* cells or knocking it down by an RNAi approach, efforts are on to gain cell biological insights on these aspects. Nevertheless, as a first pass, we have uncovered some interesting aspects of the protein based on which we speculate that UVI31+ protein may be involved in UV-damage response and repair under the regulation of light-dark cycles.

It is important to note that we still do not fully understand the transit peptide sequence motifs that are required for several thousands of proteins that are nuclear coded but are subsequently imported into organelles such as chloroplast, mitochondria and peroxisomes etc [26]. The problem is more compounded when it relates to chloroplast transit peptide signatures, which appear highly diverse [26]. Since the protein sequence showed no chloroplast-specific import tags, it is likely that UVI31+ protein reaches pyrenoid location in chloroplast by the help of an unknown interactor protein.

mRNA transcripts of *UVI31*+ show a twelve and six fold increase in expression during UV stress and dark incubation respectively in *C. reinhardtii* cells (Fig. 1). The presence of the upstream DRE elements reflects the role of these sequences in the over expression of the *UVI31*+ transcripts in the DNA damaging conditions of UV exposures [13]. The presence of DRE elements and the possible up regulation of these elements after UV exposure underscores *UVI31*+ role in UV survival response as shown in over expressing *E. coli* (Figure 2B). The cells enter into S phase of cell cycle as soon as they go into the dark phase, where in the cell cycle regulation genes including that of *UVI31*+ get up regulated. Thus the presence of MCB and SCB elements in the upstream region explains the over expression of *UVI31*+ transcripts during transition to dark (Figure 1 & 5C) when the cell prepares for division cycles and enters into G1-S phase of cell cycle.

Pyrenoid localization of UVI31+ is interesting, but also intriguing. NMR is a powerful, high structural resolution technique that is used to demonstrate the interaction between protein-protein and protein with other target moieties. Moreover, we probed UVI31+ binding to pyrenoid at amino acid residue level resolution to map the UVI31+ protein domain that interacts with pyrenoid with an intention of designing appropriate genetic perturbations, *in vivo*. We therefore used this ability of NMR to decipher the interaction of the labeled protein with the isolated Pyrenoid from cells. Specific interaction of UVI31+ protein via its N-terminal flexible domain with exogenously added pyrenoid fractions (Figure 6 & 7) was observed and is consistent with its localization to pyrenoids *in vivo*. Recent studies have clearly demonstrated that pyrenoid in *C. reinhardtii* chloroplasts are associated with processing of RNA stress granules [12] and may also be de novo sites of RNA translation and DNA association [11]. However the functional and physiological implications of these findings are unclear. Studies hint towards the fact that pyrenoid proteome represents a dynamic hub of regulation involving carbon concentrating mechanism associated proteins as well as regulatory components associated with RNA and DNA processing [11], [12]. Perhaps UVI31+ protein is a part of such protein network. We speculate that its DNA endonuclease activity might form a part of hitherto unknown DNA repair/modulation in pyrenoid fraction. If pyrenoids play a role during chloroplast cup division, UVI31+ protein component might be a regulator of chloroplast septation, as it specifically localizes to pyrenoids in dark phase when cells go through successive divisions. Our current efforts are focused on uncovering the functional significance of these changes in UVI31+ protein dynamics. Dynamic nature of protein association and dissociation with pyrenoids is already well exemplified: RuBisCo shuttles between pyrenoids and stroma in *C. reinhardtii* chloroplasts as a function of light and dark cues [27]. There are indications that lipid biogenesis ensues in pyrenoids when cells are subjected to lipogenic conditions, suggesting that lipogenic proteins/enzymes might be dynamically recruited there [28]. It is very likely that pyrenoids in *C. reinhardtii* chloroplasts are intensely active dynamic “hubs” of cellular regulation.

## Materials and Methods

### Strains, Cell Cultures

All the *C. reinhardtii* strains were obtained from Chlamy Database Duke University, Durham, North Carolina, USA. Cells were grown in TAP (Tris acetate phosphate) or TP (Tris phosphate) culture medium [29] and supplemented with arginine for strain CC3395.

### Growth Conditions

We inoculated *C. reinhardtii* colonies from TAP plates into 150 mL of TP liquid medium, grew the culture with continuous shaking in light-dark (12 hr: 12 hr X for 3 or 4 days) until the cells reach mid-log phase and get synchronized, which we consider as primary culture or inoculum. We used this inoculum in 600 mL of TP medium, continued the growth in a regime of 12 hr light and by 12 hr dark with continuous shaking (Bernstein, 1960). Cultures are grown photo autotrophically at an intensity of 90 µmol/m^2^/s. Light intensity was measured using LI-250A light meter from LI –COR Biosciences. When the cells were harvested for RNA analyses, the culture had reached mid-log phase of growth with cell density of about ∼ 8×10^5^ to 1.0×10^6^ cells/ml. All analyses were performed on four types of samples growing at light-dark (12 hr: 12 hr) regime from where the cultures were retrieved at 6 hr of growth in light (Mid-light; ML), 12 hr of growth in light (Complete-light; CL), 6 hr of growth in dark (Mid-dark; MD) and 12 hr of growth in dark (Complete-dark; CD).

### UV Dosage

Cells grown in continuous light were exposed to UV-C light (4 J/m^2^/s) for 40 s, followed by incubation in dark for 1 hr and harvesting the cells for RT-PCR (Fig. 1) or immunofluorescence assay (Fig. 3). Cell viability in this condition was about 70%.

One and half hour prior to their harvesting, cells belonging to ML, CL, MD and CD samples were exposed to UV-C Light (4 J/m^2^/s) for 10 s, reverted back to their pre-UV conditions of growth and incubated for an additional 1.5 hr [30]. Thereby, this protocol generated UV-treated samples that were exposed to a short pulse of UV during ML, CL, MD and CD incubations. There was no measurable cell lethality observed in any sample following UV treatment in this protocol. Cell viability following UV-C exposure was more than 90%. UV-C light intensity was measured using ILT 77 germicidal radiometer from International Light Technologies. Cells were harvested for RT-PCR and immunofluorescence assay (Fig. 5).

### RT-PCR, Cloning of Wild-type *uvi31*+ Coding Sequences, Sequence Analysis and Alignments

The *C. reinhardtii UVI31*+ coding sequence was amplified by PCR using cDNA as template. cDNA was prepared from DNase-treated total RNA prepared from logarithmic phase culture as described [31]. Gene specific primers (Fw-5′GCGGATCCATGAGAGGATCGCATCAC 3′; and Rv-5′ TTACTGCTCTGCCGGTGTCTTTG 3′) were used for PCR amplification. In order to semi-quantify the transcript, *C. reinhardtii* actin gene was used as an internal reference. The primers used for amplifying actin cDNA were; Fw-5′ATGATCACCATCGGCAACG3′; and Rv-5′TGTTGTTGTAGAGGTCCTTGCG 3′. The *UVI31*+ cDNA was cloned into two N-terminal His-tagged vectors; pQE30-UA and pET28a. pQE30-UA is a pre-linearized expression vector (Qiagen) which has a U-overhang in its multiple cloning site and allows direct cloning of PCR products, which have an A-overhang. In case of cloning in pET28a (Novagen), the *UVI31*+ cDNA was amplified using Pwo DNA polymerase (Roche), which produces blunt-ended PCR products. The cDNA was cloned into the *Nde*I cleaved and blunted (using DNA polymerase I large Klenow fragment from Promega) pET28a expression vector. The ligated products were used to transform competent *E. coli* BL21 (DE3) cells. Recombinant clones were selected on LB plates containing ampicillin (100 µg/ml) or kanamycin (50 µg/ml) as per requirement. The presence and orientation of the insert in the vector was confirmed by colony PCR using a combination of plasmid specific and gene specific primers (data not shown). It was further confirmed by sequencing. The resulting plasmids were named pRKM201 and pRKM202, respectively.

### Expression and Purification of UVI31+

*E. coli* strain BL21 (DE3) harboring the vector pRKM201 or pRKM202 was grown in LB medium containing ampicillin (100 µg/ml) or kanamycin (50 µg/ml) to an absorbance A600 of 0.5 at 33°C, followed by induction with 1 mM IPTG at 25°C for 4 h. Cells were collected by centrifugation, resuspended in lysis buffer [50 mM sodium phosphate (pH 7.6), 50 mM NaCl, 1 mM PMSF, 5 mM Imidazole, 2% Tween 20, and 1 mg/ml lysozyme] and incubated on ice for 30 mins. Cells were disrupted by ultrasonication. The cell debris was removed by centrifugation (15,000 rpm for 20 min at 4°C). UVI31+ protein was purified from the resulting supernatant using Ni-NTA (Ni2+-nitrilotriacetate) agarose (Qiagen, Hilden, Germany). His6-tagged UVI31+ was eluted with 250 mM imidazole in 50 mM sodium phosphate (pH 7.6), 50 mM NaCl. The eluted fractions were dialyzed overnight against 50 mM sodium phosphate (pH 7.6), 50 mM NaCl at 4°C. The protein was further purified by gel filtration using a Sephadex G75 column (GE healthcare, USA) equilibrated with 50 mM sodium phosphate (pH 7.6), 50 mM NaCl. N-terminal amino acid sequence analysis was performed by the Proteomics International Pty Ltd, Australia. UVI31+ was quantitated by Bradford with bovine serum albumin as a standard [32], and by measuring absorbance at 280 nm. Typical yields were 25 to 30 mg/l of culture.

### UV Survival Test

*E. coli* BL21 (DE3) cells over-expressing UVI31+ protein (*E. coli* pRKM201) and control strain (*E. coli* pQE30) were grown in LB medium containing ampicillin (100 µg/ml) to an absorbance A600 of 0.3 at 33°C, followed by induction with 1 mM IPTG at 33°C for 1 h. Cells were harvested and resuspended in an equal volume of 0.85% saline. Aliquots (100 µl) of appropriate dilutions (in saline) were spread on LB agar plates and immediately exposed to UV light from a TUV15Wgermicidal Mercury vapor lamp (Philips). The intensity of irradiation was 0.70 J/m^2^/s^1^. Survivors were determined after an overnight incubation at 37°C. All procedures were carried out in dark to prevent photo-reactivation.

### Endonuclease and Zymogram Assay

For agarose gel assay Each reaction mixture (20 µl total volume) contained 300 ng of negatively supercoiled pBR322 DNA, non-irradiated or irradiated at the indicated dose, 50 mM sodium phosphate (pH 7.6), 50 mM NaCl, 1 mMMgCl_2_ and UVI31+, followed by incubation at 37°C for 30 min. Reaction was stopped by adding 5 µl of stop solution (10% glycerol, 0.005% bromophenol blue, 0.1% SDS). DNA samples were analyzed by electrophoresis at 3V/cm for 4 hr on a 0.8% agarose gel in Tris-acetate-EDTA buffer (40 mM Tris-acetate, 1 mM EDTA pH 8.0). The gel was stained in ethidium bromide solution (0.5 µg/ml) for 30 min, and finally visualized in a UV transilluminator.

The DNAse zymogram was performed by running the gel on an SDS page containing 0.1 mg/ml denatured Salmon sperm DNA. The protein in the gel was allowed to renature in PEB buffer [Phophate buffer (pH 7.6), 5 mM NaCl, 0.5 mM EDTA] for 48 hrs during which the gel was washed three times. The gel was then incubated in 0.05% EtBr solution, followed by visualization of the gel on a UV transilluminator. The endonuclease activity was observed as dark band reflecting absence of DNA due to DNAse activity on an ErBr fluorescing background caused by DNA wherever endonuclease was absent.

### mRNA Expression Analysis

Cells were pelleted from ML, CL, MD and CD cultures; RNA was extracted using Trizol reagent following the guidelines provided (Bangalore Genei). The RNA concentrations were determined with Nanovue plus from GE health care, Life sciences and the integrity of the RNA was confirmed by Agarose gel electrophoresis. mRNA was further isolated using an oligotex mRNA isolation kit (Qiagen). In each reaction, 50 ng of mRNA was used for cDNA synthesis following the manufacturer’s instructions (RT-PCR kit, Bangalore Genei) using poly(dT) primers. This cDNA was amplified using kappa master mix using the *UVI31*+ gene specific primers shown above. Actin cDNA amplification was used as an internal control in each sample. ‘No template controls’ (NTC) and ‘no-RT’ controls were included in all runs to exclude potential DNA contamination. RT-PCR products analyzed by agarose gel electrophoresis matched with expected RT-PCR product of *UVI31*+ rather than its genomic PCR product. We sequenced RT-PCR products to validate the nature of products under study. Following gel analyses, RT-PCR DNA band intensities were measured by Image J intensity measurement program. The intensity of *UVI31*+gene transcript was expressed by its RT-PCR DNA band intensity divided by that of actin band intensity. Fold enhancement of transcript is computed by comparing such normalized values.

### Isolation of Cell Wall Shells and Pyrenoids

Cell wall shells were isolated from a one-liter culture of CC125 grown in TAP medium under continuous light by the mechanical disruption method followed by Imam et al (1985) [17]. The cells were deflagellated by pH-shock method [33]. Cell wall shell pellet was obtained from deflagellated cells after glass bead mediated mechanical disruption, followed by high-speed centrifugation.

Pyrenoids were isolated according to the method described by Kuchitsu et al (1988) using one-liter culture of cell wall less mutant (CC3395) cells [16]. Themethod essentially involved sonication of the cellwall less mutant, treatment of the lysate with 1% NP40 detergent, followed by density gradient centrifugation that pelleted pyrenoid-rich fraction.

### Western Blotting

Proteins were resolved by SDS-PAGE, transferred to a nitrocellulose membrane (GE health care), followed by incubation with primary antibodies for 1 hr, according standard conditions. The primary antibody (1∶1000) was raised in rabbit, purified by gel exclusion chromatography followed by affinity purification using purified UVI31+ protein. The membrane was probed by secondary antibodies (1∶1000) according to the standard protocol, followed by developing the blot by DAB reagent(metal enhanced) (Amersham, Piscataway, NJ). Purified primary antibody was neutralized by incubating it with UVI31+ protein (100 µM) overnight in PBST-BSA buffer. Such neutralized antibody was used for control experiments. Anti-tubulin antibody incubated with UVI31+ protein was used as a control to show that such neutralization protocol does not reduce its titer.

### Immunofluorescence

Standard protocol of immunostaining was followed [34]. Briefly, immunofluorescence staining was carried out by immobilizing the cells on a 0.1% poly-lysine coated slide, followed by fixation with chilled methanol (10 min) and acetone (6 min). The cells were blocked by immersion for one hour in PBST-3% BSA blocking buffer. The slides were further incubated with antiUVI31+ antibody (1∶200), followed washing (thrice of five min each) in the blocking buffer and incubation with anti-rabbit secondary antibody tagged with FITC (1∶200). The cells were observed in a Ziessepi fluorescence or Zeiss 510 laser confocal microscope after immersion in a DAPI containing mounting medium.

### NMR Studies on UVI31+ and Pyrenoid

For NMR experiments, uniformly ^15^N-labeled protein (u-^15^N-UVI31+) sample was produced in minimal (M9) media supplemented with ^15^NH_4_Cl as the sole source of nitrogen [14]. Purified UVI31+ protein (200 µM) was added to pyrenoids fraction prepared from 100 ml *C. reinhardtii* culture and incubated for 1 hour at 4°C. Free UVI31+ protein and UVI31+ bound to pyrenoids fraction were prepared in a mixed solvent of 90% H_2_O and 10% ^2^H_2_O [50 mM sodium phosphate (pH 6.4), 100 mM NaCl]. NMR experiments were recorded at 298 K on a Bruker Avance 800 MHz NMR spectrometer equipped with a 5 mm triple-resonance cryogenically cooled probe at 298 K. Experiments recorded with u-^15^N UVI31+ included sensitivity enhanced 2D [^15^N-^1^H]-HSQC using water-flip-back for minimizing water saturation. ^1^H chemical shifts were referenced to the external standard 2,2-dimethyl-2-silapentene-5-sulfonates (DSS). ^15^N and ^13^C chemical shifts were calibrated indirectly from DSS.

## Supporting Information

Figure S1 SDS-PAGE analysis of expression and purification of UVI31+ protein. Molecular weight marker (lane 1); Total cell lysate of *E. coli* BL21 cells over expressing UVI31+ in absence (lane 2) and presence (lane 3) of IPTG; Purified UVI31+ protein (lanes 4 & 5); Western blot analysis of purified UVI31+ protein probed with anti-Histidine antibody (lane 6). Coomassie Brilliant Blue stained gel (lanes 1–4); Silver nitrate stained gel (lane 5).
(TIF)

Figure S2 UVI31+ immunofluorescence confocal image stacks (twelve stacks arranged from left to right in three rows) of CC3395 control (A) and 160 J/m^2^ UV treated cells (B).
(TIF)

Figure S3 Western blot analysis using combined anti UVI31+ & anti tubulin antibody mixture on the standard UVI31+ protein (lane 1), whole cell extracts from control (lane 3) and UV-treated cells(160 J/m^2^) (lane 4). The dimer and monomer positions from UVI31+ are shown at 24 and 13.4 kDa respectively. Whole cell extract lanes (3 & 4) show only tubulin signal at expected location.
(TIF)

Table S1 Percent identity of UVI31+ with BolA-like proteins from other organisms
(DOCX)
