# Supplementary figures and images for: UVI31+ Is a DNA Endonuclease That Dynamically Localizes to Chloroplast Pyrenoids in C. reinhardtii
Source: PLoS One. 2012 Dec 17;7(12):e51913. doi: 10.1371/journal.pone.0051913 (PMC3524116; doi:10.1371/journal.pone.0051913)

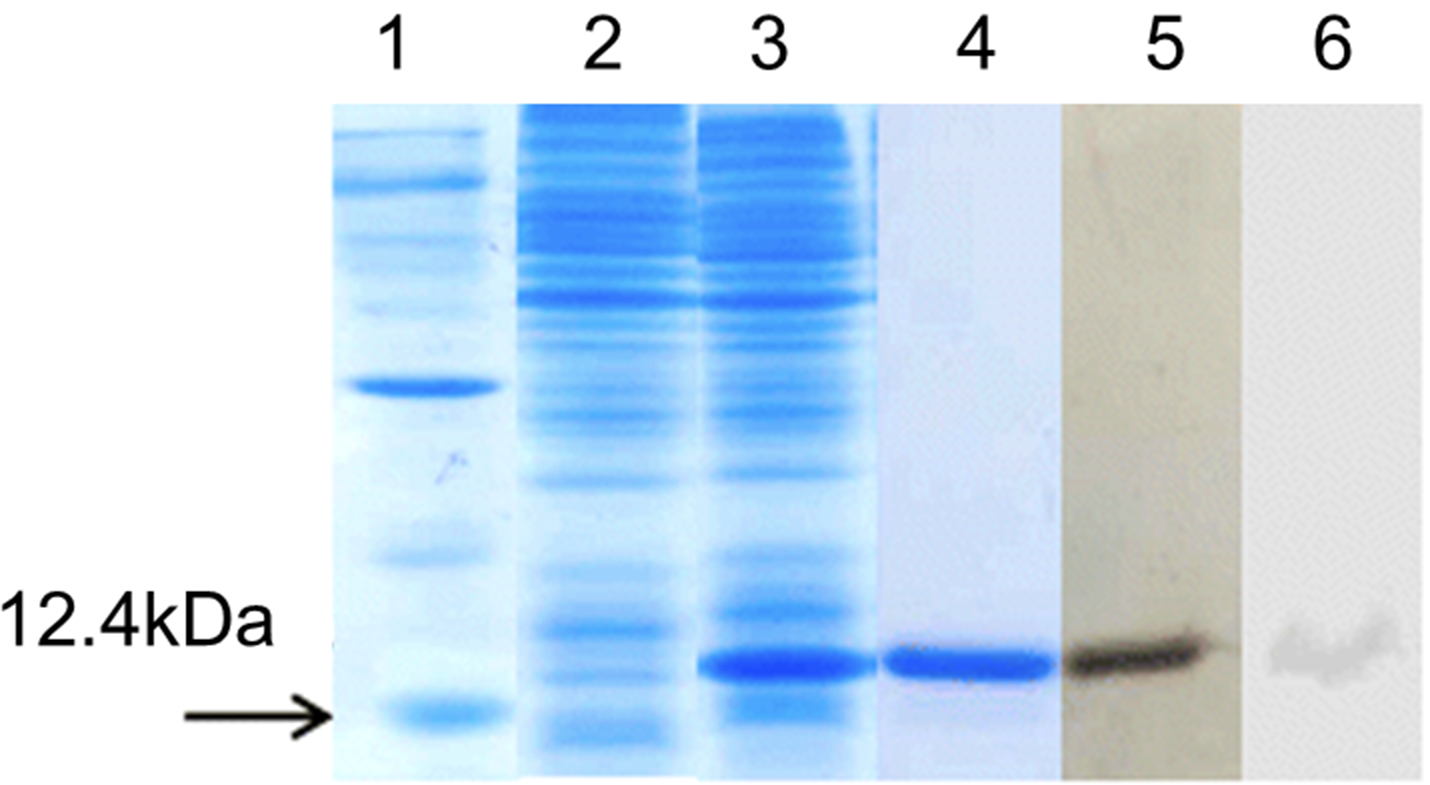

Supplement: Figure S1 — SDS-PAGE analysis of expression and purification of UVI31+ protein. Molecular weight marker (lane 1); Total cell lysate of E. coli BL21 cells over expressing UVI31+ in absence (lane 2) and presence (lane 3) of IPTG; Purified UVI31+ protein (lanes 4 & 5); Western blot analysis of purified UVI31+ protein probed with anti-Histidine antibody (lane 6). Coomassie Brilliant Blue stained gel (lanes 1–4); Silver nitrate stained gel (lane 5). (TIF) [file pone.0051913.s001.tif]

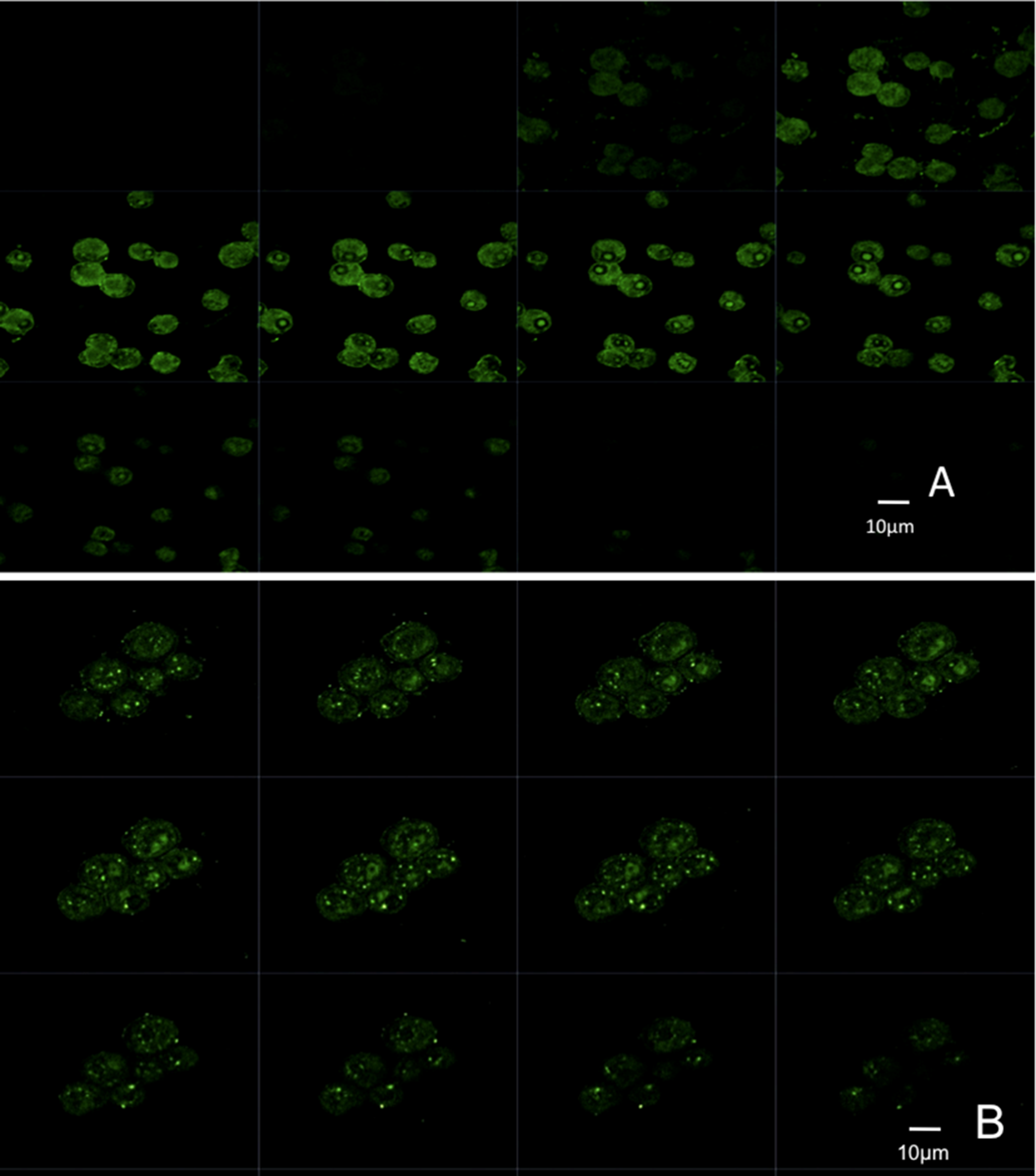

Supplement: Figure S2 — UVI31+ immunofluorescence confocal image stacks (twelve stacks arranged from left to right in three rows) of CC3395 control (A) and 160 J/m2 UV treated cells (B). (TIF) [file pone.0051913.s002.tif]

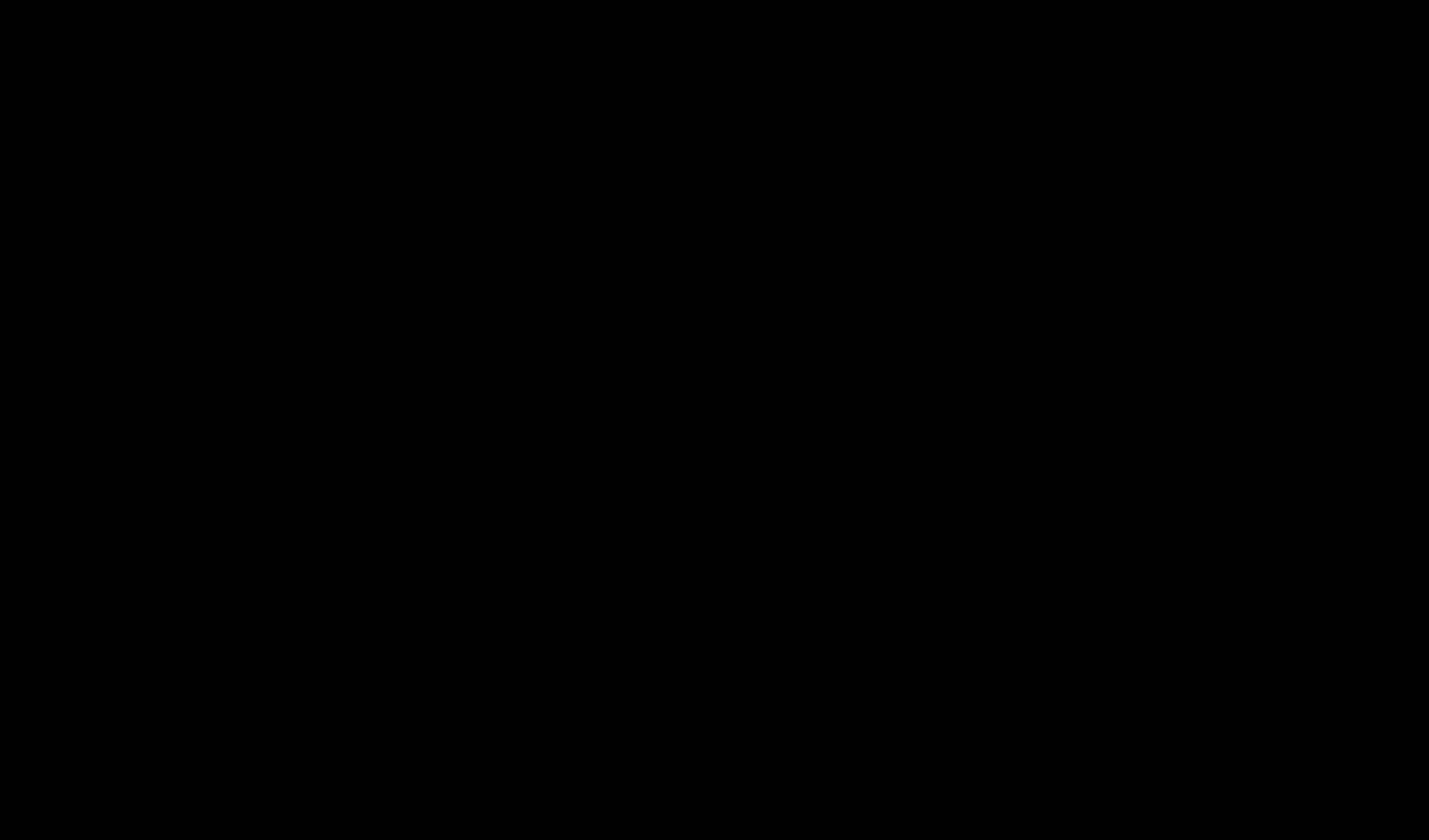

Supplement: Figure S3 — Western blot analysis using combined anti UVI31+ & anti tubulin antibody mixture on the standard UVI31+ protein (lane 1), whole cell extracts from control (lane 3) and UV-treated cells(160 J/m2) (lane 4). The dimer and monomer positions from UVI31+ are shown at 24 and 13.4 kDa respectively. Whole cell extract lanes (3 & 4) show only tubulin signal at expected location. (TIF) [file pone.0051913.s003.tif]
