# Supplementary material for: UVI31+ Is a DNA Endonuclease That Dynamically Localizes to Chloroplast Pyrenoids in C. reinhardtii
Source: PLoS One. 2012 Dec 17;7(12):e51913. doi: 10.1371/journal.pone.0051913 (PMC3524116; doi:10.1371/journal.pone.0051913)
Supplement: Table S1 — Percent identity of UVI31+ with BolA-like proteins from other organisms (DOCX) [file pone.0051913.s004.docx]

**Supplementary Table 1:** Percent identity of UVI31+ with BolA-like proteins from other organisms

| **No.** | **Organsim** | **% Identity** | **Length (aa)** | **Accession number** |
| --- | --- | --- | --- | --- |
| 1 | *C. reinhardtii* | 100 | 100 | XP_001702905 |
| 2 | *C. albicans* | 48 | 121 | XP_722220 |
| 3 | *A. clavatus* | 43 | 134 | XP_001272395 |
| 4 | *S. cerevisiae* | 42 | 110 | NP_075206 |
| 5 | *T. thermophila* | 42 | 117 | XP_001471032 |
| 6 | *S. pombe* | 40 | 102 | CAB_16898.1 |
| 7 | *A. fumigatus* | 40 | 135 | XP_746780 |
| 8 | *O. sativa* | 37 | 102 | ABR_25678 |
| 10 | *R. norvegicus* | 36 | 137 | NP_001065244 |
| 11 | *M. musculus* | 36 | 137 | NP_081251 |
| 12 | *B. melitensis* | 36 | 102 | NP_538963 |
| 13 | *H. sapiens* | 35 | 137 | AAH_63405 |
| 14 | *B. taurus* | 35 | 135 | NP_001029524 |
| 15 | *E. coliK12* | 27 | 105 | NP_414969 |
